# Cost-Effectiveness Analysis of Adding Telerehabilitation to Standard Care for Ankle Sprains Compared with Standard Care Alone

**DOI:** 10.1089/tmr.2025.0010

**Published:** 2025-04-11

**Authors:** Juan Figueroa-García, Víctor Marcial Granados-García, Juan Carlos H. Hernández-Rivera, David Rojano-Mejía

**Affiliations:** ^1^ Unidad de Medicina Familiar N. 26, Órgano de Operación Administrativa Desconcentrada de la Ciudad de México Sur, Instituto Mexicano del Seguro Social, Ciudad de México, México.; ^2^ Unidad de Investigación Epidemiológica y en Servicios de Salud, Área Envejecimiento, Instituto Mexicano del Seguro Social, Ciudad de México, México.; ^3^ Unidad de Investigación Médica en Enfermedades Nefrológicas, Hospital de Especialidades, Centro Médico Nacional Siglo XXI, Instituto Mexicano del Seguro Social, Ciudad de México, México.; ^4^ Coordinación de Investigación en Salud, Instituto Mexicano del Seguro Social, Ciudad de México, México.

**Keywords:** cost-effectiveness, telerehabilitation, telehealth, ankle sprain, rehabilitation, clinical trial

## Abstract

**Background:**

Ankle sprain (AS) is a common musculoskeletal injury. While telerehabilitation is an effective treatment for various musculoskeletal conditions, evidence on its cost-effectiveness for AS is lacking.

**Methods:**

A cost-effectiveness study was conducted through a 4-week randomized controlled trial in individuals with AS. The control group (*n* = 41) received standard care, while the intervention group (*n* = 41) received standard care plus asynchronous telerehabilitation. Effectiveness was measured using the Foot and Ankle Ability Measure (FAAM) with subscales for daily living (FAAM-ADL) and sports activities (FAAM-sports). The economic evaluation used the Mexican health system’s official price list, including work incapacity costs, updated to 2024 (U.S. dollars). A one-way sensitivity analysis was also performed.

**Results:**

At 4 weeks, the intervention group showed a gain of 78 points in functionality for FAAM-ADL and 80.2 points for FAAM-sports, while the control group scored 69.1 and 61.6, respectively. When the costs of work incapacity were considered, the incremental cost-effectiveness ratio (ICER) of adding telerehabilitation per point gained in FAAM-ADL functionality was US$ −14.4 and US$ −8.5 for FAAM-sports. When work incapacity costs were excluded, the ICER was US$ −0.7 and US$ −0.4, respectively.

**Conclusions:**

Adding telerehabilitation to standard care for AS was cost-saving, achieving greater effectiveness at a lower cost. This is more evident when direct costs are considered together with the costs of work incapacity.

## Introduction

Ankle sprain (AS) is one of the most common musculoskeletal injuries, causing significant morbidity when not adequately treated.^1–4^ The most crucial therapeutic component for AS is physical rehabilitation. Although this is well known, AS is often underestimated by both physicians and patients, leading to inadequate treatment.^2,5,6^ Another characteristic aspect of AS is associated pain, which reduces physical ability for mobility or transportation (e.g., visiting the doctor or attending in-person rehabilitation sessions).^7,8^ One solution to these limitations is telerehabilitation, which has demonstrated clinical efficacy in various musculoskeletal conditions and has become an increasingly useful tool because of its potential to reduce costs and improve accessibility to care.^9,10^

Regarding the rehabilitation of ASs, it is known that inadequate rehabilitation therapy can have short- and long-term economic repercussions.^4^ However, there appears to be little or no published information on the cost-effectiveness of telerehabilitation for ASs.^7^ Telerehabilitation allows patients to receive care and perform therapeutic exercises via digital platforms, eliminating the need to travel to a rehabilitation center.

This modality not only increases comfort for the patient but may also significantly reduce the direct and indirect costs associated with treatment.^7,11^

Therefore, we hypothesize that in a cost-effectiveness analysis, adding telerehabilitation to standard care for ASs is a more effective and cost-saving measure. The objective of this research was to perform a cost-effectiveness analysis comparing the use of telerehabilitation plus standard care versus standard care alone.

## Materials and Methods

### Design

This cost-effectiveness analysis, conducted from the provider’s perspective, evaluated direct cost using data from a pragmatic randomized controlled trial. The trial compared standard treatment for ASs in primary care level versus standard treatment combined with telerehabilitation.

### Participants

The selection criteria were individuals with a diagnosis of grade I or II AS within 72 h of injury, aged between 18 and 60 years, first-time diagnosis of AS, able to use a cell phone or computer, and willing to participate in the study and sign the informed consent. The exclusion criteria were: previous history of AS, central or peripheral neurological disorders, lower limb ulcers, fibromyalgia, pre-existing ankle arthritis, history of severe injuries in the ipsilateral or contralateral lower limb, and use of oral or intravenous glucocorticoids. The study protocol was approved by the Local Health Research Ethics Committee at the institution where it was conducted (Registration No. R-2021-3609-037). The trial was also registered at ClinicalTrials.gov (ID: NCT05217173).

### Recruitment

In the information and statistics area of the medical unit where the study was conducted, information was sought daily from patients diagnosed with ASs who were initially seen by the family physician. Both family medicine specialists and patients belonged to the same medical unit. Patients were contacted by phone and invited to participate in the study. If they agreed to participate, a personal evaluation of ankle functionality and other relevant data was conducted the next day.

### Intervention

#### Control group

Participants in this group received the standard care provided by a family medicine specialist at the primary care level. The medical care consisted of the standard therapeutic approach that the treating physician considered appropriate for the patient. The specific therapeutic actions taken were not investigated.

#### Intervention group

Participants received standard care plus telerehabilitation. Telerehabilitation consisted of instructions to perform rehabilitation exercises available in pre-recorded videos hosted on a digital platform (MoodleCloud™), accessible 24 h a day. Instructions also included the duration of exercise sessions (30 min/day, 5 days/week, for 4 weeks). The ankle rehabilitation exercises were divided into exercises for weeks 1 and 2, the exercises focused on mobility/stretching and proprioception, and in weeks 3 and 4, they focused on mobility/stretching and strength. The exercise program was based on recommendations from national and international clinical practice guidelines.

### Outcome measures

The outcome measures included assessment of ankle functionality and pain conducted at baseline and weekly during the 4-week rehabilitation program; disability days were quantified at the end of the program. The primary outcome measure was patient-perceived ankle functionality, assessed in both the intervention group (IG) and control group (CG) using the Foot and Ankle Ability Measure (FAAM). This instrument consists of two subscales: Activities of Daily Living (ADL) with 21 items and Sports Activities (SA) with 8 items. Each item is scored on a 5-point Likert scale (4 to 0) from “no difficulty at all” to “unable to do,” with N/A responses not counted. The total item scores, ranging from 0 to 84 for the ADL subscale and 0 to 32 for the SA subscale, were transformed into percentage scores. Higher scores represent higher levels of function for each subscale, with 100% indicating no dysfunction.^12^ The secondary outcomes included pain and days of work incapacity. Pain was assessed using the Visual Analog Scale (VAS) for pain, which consists of a 10-cm horizontal line with extreme expressions of pain at each end; the left end of the line corresponds to 0, indicating no pain, while the right end represents the maximum pain perception with a score of 10. The total days of work incapacity were quantified at the end of the 4-week study period. Additionally, the total minutes of exercise performed through telerehabilitation were measured only in the intervention group (IG), using a self-reporting method.

### Data collection for cost-effectiveness

The economic evaluation consisted of an analysis based on the clinical trial. The unit prices of goods and services were obtained from the official price list of the health service provider, a government agency.^13^ Costs were updated to 2024 Mexican pesos with national index of consumer prices and converted to U.S. dollars using an exchange rate of 18.15 Mexican pesos per dollar (average exchange rate for the month of June 2024 according to the Bank of Mexico). Effectiveness (health gain) was measured by ankle functionality using the FAAM, with independent analysis for each subscale.

For both treatment alternatives, the costs considered were: (1) days of incapacity due to the diagnosis of AS; for each patient, a cost was assigned for each day of incapacity, based on the average salary in Mexico, according to the official report of the service provider. In the end, the cost was averaged by the number of days of disability; (2) average cost of the number of medical consultations for sprained ankle at the primary care level; (3) average costs of the number of radiographs performed of the affected ankle; and (4) average cost of analgesic medications and Nonsteroidal anti-inflamatory drugs (NSAIDs) for the treatment of sprained ankle, and each box of medication was given a cost.

For the IG, additional costs included (1) annual subscription cost (small plan with 1 GB of storage) of MoodleCloud^TM^ application services, (2) video production costs, (3) cell phone equipment, and (4) internet service; the costs for the IG were averaged among the number of group members.

### Sample size

The findings of a study that assessed the impact of rehabilitation on ankle functionality (evaluated using FAAM) served as a reference. The difference recorded between pre- and post-rehabilitation was 9.1 in the FAAM-ADL subscale, with a standard deviation of 11.07.^14^ For the calculations, a power of 90%, loss 20%, and an alpha level of <0.05 were used, leading to a requirement of 41 participants per group.

### Randomization and group allocation

Participants were randomly allocated to either the IG or the control group (CG) using a single randomization list created in Excel for macOS. To generate this list, the number “1” (representing IG) and the number “2” (representing CG) were entered into cells in a column, each repeated 41 times. In a parallel column, random numbers were generated, fixed, and then sorted in descending order. The order of this sorted sequence was utilized to assign participants to groups based on the first column. The randomization sequence remained confidential under the control of the principal investigator, and group assignments were communicated verbally to collaborators as each patient joined the study.

### Blinding

Due to the necessity for participants to be informed about the instructions pertaining to their respective treatment groups, and because the initial evaluators were responsible for providing these instructions, it was not feasible to blind both parties at the outset. In subsequent weekly evaluations, different assessors, who were unaware of the participants’ treatment allocations, performed the assessments. The data analyst maintained blinding throughout the duration of the study.

### Statistical analysis

Data were analyzed in accordance with the intention-to-treat principle. Missing data from participants who did not complete follow-up assessments were imputed using predictive mean matching. The Shapiro–Wilk test was conducted to assess the normality of the data distribution. In instances where the normality assumption was violated, the median and interquartile ranges were reported. Comparison of the results of the dependent variables was performed with the Mann–Whitney *U* test, and weekly data within each group were compared. The associations between variables and the distribution of results across groups were assessed using a chi-square test, with *p* < 0.05 deemed indicative of statistical significance. Data statistical analysis was carried out utilizing SPSS V.26 software and Excel for MacOS for the economic analysis.

## Results

**FIG. 1.**
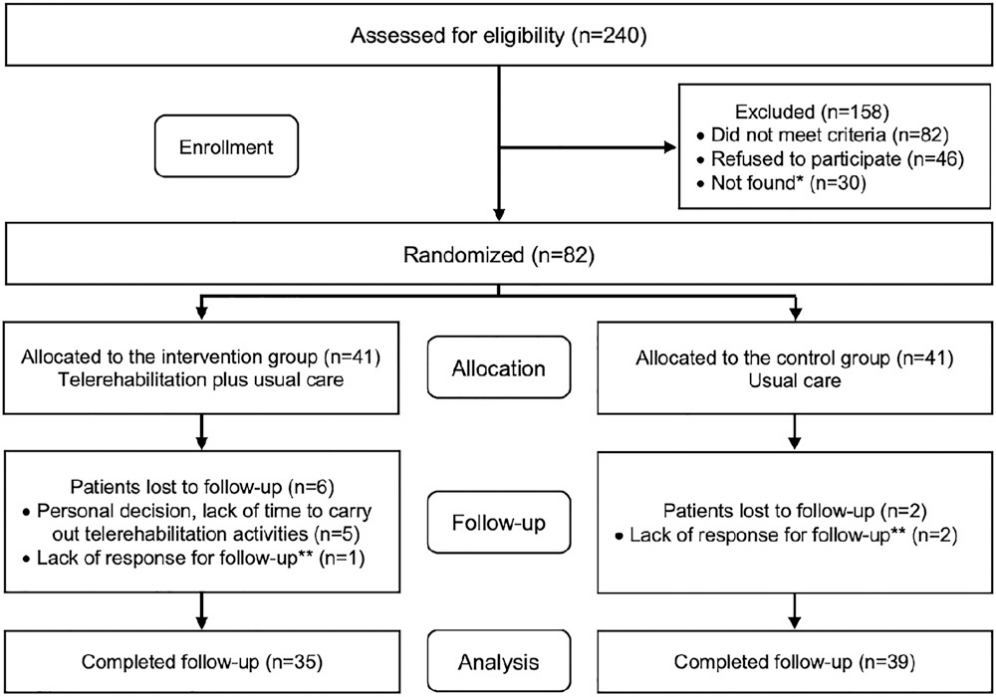
Flow diagram of the study. *Patients who did not answer the initial phone call to receive an invitation to the study. **Patients who did not attend appointments for further evaluations and did not answer telephone calls for contact.

Recruitment of participants was from February to October 2022. The study sample consisted of 82 participants, 41 in the IG and 41 in the CG (Fig. 1).

The mean age was similar in both groups. A statistically significant association was found only between educational level and the treatment group (*p* < 0.05). Both groups had a higher concentration of patients with a high school education (43.9% in the CG and 41.5% in the IG). The CG also had a higher mean body mass index. Just over half of the patients in the CG were diagnosed with a grade II AS (51.2%), compared with 70% in the IG (Table 1).

**Table 1.** Demographic and Clinical Characteristics of the Sample

Variable	Intervention (*n* = 41)	Control (*n* = 41)
Age, mean (SD)	35.22 (10.3)	36.8 (11.3)
Sex, frequency (%)		
Female	18 (44)	22 (54)
Education, frequency (%)^a^		
Primary	1 (2.4)	0
Secondary	7 (17.1)	17 (41.5)
High school	17 (41.5)	18 (43.9)
Bachelor’s or more	16 (39)	6 (14.6)
BMI, mean (SD)	26.9 (5.2)	28.6 (6.2)
Ankle sprain grade, frequency (%)		
Grade I	20 (48.8)	12 (29.3)
Grade II	21 (51.2)	29 (70.7)

^a^ Chi-square with *p*-value of <0.05.

BMI, body mass index; SD, standard deviation.

At the end of 4 weeks, the IG exhibited fewer days of incapacity, greater ankle functionality in both FAAM subscales (ADL and Sports), and lower pain perception (measure with VAS). These comparisons were statistically significant (*p* < 0.05) (Table 2). Cost determination was conducted in Mexican pesos. Across all categories considered, higher costs were observed in the CG.

**Table 2.** Differences Between Groups in Days of Work Incapacity, Ankle Functionality Measured by FAAM and VAS, Health Care Resource Costs Utilized, and Adherence Self-Reported

	Intervention (*n* = 41)		Control (*n* = 41)	
Measure	Median	IQR	Median	IQR
Incapacity days for work^a^	7	5–13	13	7–15
FAAM score ADL^b^				
Baseline	15.2	7–27.1	13	5.9–26.3
Week 4^a^	93.2	86.2–95.8	82.1	76.8–88.3
FAAM score sports				
Baseline	2	0–9.3	6.2	0–9.1
Week 4^a^	82.2	67.4–89.2	67.8	57.2–73.5
VAS-pain				
Baseline	9	8–9	9	8–9.5
Week 4^a^	1	0–2	2	1–3

Health care resource costs per person^b^	Mean	CI 95%	Mean	95% CI
Consultations	$147.2	$150 to 209	$179.9	$150 to 209
X-rays	$44.5	$37 to 56	$53	$43 to 62
Medications	$1.2	$1 to 1.4	$1.38	$1.1 to 1.5
Incapacity days for work	$233.9	$191 to 276	$354.3	$298 to 410
Intervention cost	$32.7	$32.6 to 32.8	—	—

	Minutes	%		
Total minutes of self-reported rehabilitation exercise^c^	17,171	69.8		

^a^ *p*-Value of <0.05 with the Mann–Whitney *U* test between the control and intervention groups.

^b^ Costs in U.S. dollars.

^c^ The total expected rehabilitation exercise time for the 41 people in the intervention group was 24,600 min (30 min/day, 5 days/week, for 4 weeks), and the percentage is the cumulative self-reported minutes of the entire intervention group during the 4 weeks.

ADL, Activities of Daily Living; CI, confidence interval; FAAM, Foot and Ankle Ability Measure; IQR, interquartile range; VAS, Visual Analog Scale for pain.

Four main outcomes were identified: the incremental cost-effectiveness ratio (ICER) of ADL and SA considering costs of incapacity days and therefore work absenteeism generated by AS, and the ICER of ADL and SA without considering work incapacity days costs, only taking into account health care costs for the CG and IG resources for the telerehabilitation-assigned group. The ICERs for both FAAM subscales that considered incapacity for work costs were more dominant, showing higher effectiveness and lower cost (for ADL, US $14.4 and US $8.5 for SA), whereas those considering only health care services costs, without incapacity work cost, were also dominant, but with a lower incremental ratio (ADL, US $0.7 and SA, US $0.4) (Table 3).

**Table 3.** Cost-Effectiveness Analysis of Telerehabilitation Versus Usual Care on Ankle Functionality

Treatment alternative	Mean effectiveness	Mean cost^a^	CER (threshold)	ICER
Cost-effectiveness of ankle functionality with incapacities costs in ADL				
A	62.6	562.3	9.0	—
B	71	441.0	6.2	−14.4
*Cost-effectiveness of ankle functionality with incapacities costs in Sports Activities*				
A	57.4	562.3	9.8	—
B	71.7	441.0	6.2	−8.5
*Cost-effectiveness of ankle functionality without incapacities costs in ADL*				
A	62.6	223.8	3.6	—
B	71	217.6	3.1	−0.7
*Cost-effectiveness of ankle functionality without incapacities costs in Sports Activities*				
A	57.4	223.8	3.9	—
B	71.7	217.6	3.0	−0.4

^a^ Costs in U.S. dollars.

A, standard care; ADL, Activities of Daily Living from FAAM; B, telerehabilitation plus standard care; CER, cost-effectiveness ratio; ICER, incremental cost-effectiveness ratio; Sports Activities, Sports activities from FAAM.

**FIG. 2.**
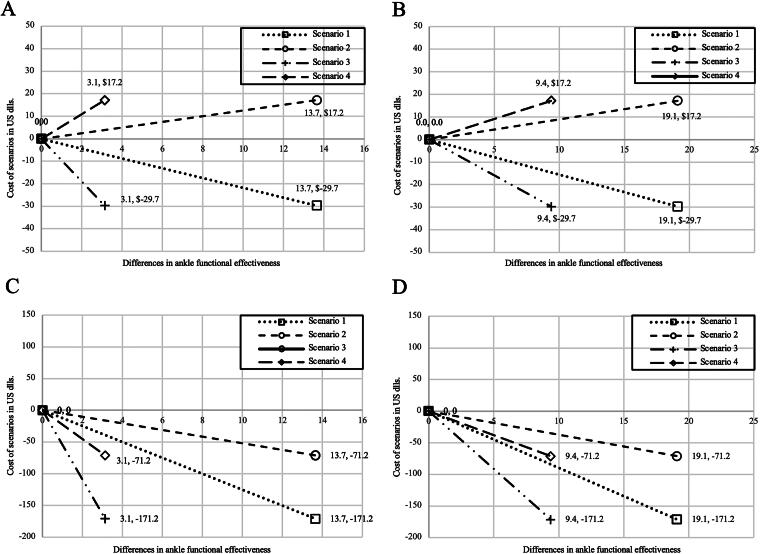
Comparison of incremental cost and effectiveness scenarios in FAAM ADL and Sports Activities. **(A)** Comparison of incremental cost and effectiveness scenarios in FAAM ADL, without incapacity days costs. **(B)** Comparison of incremental cost and effectiveness scenarios in FAAM Sports Activities, without incapacity days cost. **(C)** Comparison of incremental cost and effectiveness scenarios in FAAM ADL, with incapacity days costs. **(D)** Comparison of incremental cost and effectiveness scenarios in FAAM Sports Activities, with incapacity days costs. *Costs in U.S. dollars. ADL, Activities of Daily Living; FAAM, Foot and Ankle Ability Measure. Base case: Corresponds to standard treatment, with average cost and average effectiveness. Scenario 1: Incremental differences in costs of telerehabilitation plus standard treatment with higher effectiveness and lower cost. Scenario 2: Incremental differences in costs of telerehabilitation plus standard treatment with higher effectiveness and higher cost. Scenario 3: Incremental differences in costs of telerehabilitation plus standard treatment with lower effectiveness and lower cost. Scenario 4: Incremental differences in costs of telerehabilitation plus standard treatment with lower effectiveness and higher cost.

For the FAAM ADL subscale (Fig. 2A) and FAAM SA (Fig. 2B) without including incapacity days costs, the telerehabilitation plus standard care alternative presented two scenarios (better scenario and intermediate scenario 2) where it maintained better functionality and lower cost (dominant alternative) than the base scenario (standard treatment), and two others where it loses this economic advantage, meaning they are equally or more costly, with equal or lower effectiveness (dominated alternative). Meanwhile, in the sensitivity analysis that included incapacity days’ costs, the scenarios changed for both FAAM ADL (Fig. 2C) and FAAM SA (Fig. 2D). The four proposed scenarios with telerehabilitation plus standard care maintained higher effectiveness and lower cost (dominant alternative) than the base scenario (standard treatment).

## Discussion

The primary objective of this article was to conduct a cost-effectiveness analysis of a telerehabilitation intervention for ASs combined with standard care, compared with receiving only standard care. Overall, patients assigned to the telerehabilitation plus standard care group (IG) exhibited significant clinical improvements, characterized by a greater and more rapid recovery of ankle functionality, fewer days of work incapacity, and reduced pain perception. However, these benefits were more pronounced in patients with grade II sprains.

The main economic findings indicated that, upon performing the cost-effectiveness analysis, the treatment alternative that included telerehabilitation was found to be dominant, meaning it was cost-saving, when considering the four types of analyses conducted (FAAM ADL with and without incapacity costs, and FAAM SA with and without incapacity costs).

In our literature search, we did not find reports comparing telerehabilitation for ASs with standard care, making it challenging to contrast our results regarding cost-effectiveness in ASs; however, there are reports comparing the cost-effectiveness of telerehabilitation for other conditions (including orthopedic disorders) against traditional face-to-face rehabilitation.^8,15–20^

Our results appear consistent with some studies analyzing the cost-effectiveness of care for orthopedic conditions, where telerehabilitation demonstrated equal or superior clinical effectiveness and was found to be equally or less costly.^15,16,21^ However, the referenced studies differ from ours in several respects; one significant difference is that the mentioned studies utilized synchronous telerehabilitation via video consultations, whereas our study was conducted with prerecorded videos, providing only instructions to carry out the rehabilitation exercise plan from our program. Another difference is that our study did not compare telerehabilitation with traditional face-to-face rehabilitation; instead, telerehabilitation was compared solely with standard care (CG) provided by primary health care physicians, and it was not possible to determine if any type of rehabilitation activity was offered or indicated.

Regarding the results from the sensitivity analysis, when incapacity costs were not considered, the alternative including telerehabilitation maintained its advantage (lower cost and greater effectiveness) in two out of the four scenarios (dominant treatment alternative). Conversely, when incapacity costs were included, the telerehabilitation alternative exhibited advantages in all proposed scenarios, with lower costs and higher effectiveness (dominant treatment alternative). Therefore, it should be noted that while it may not be a cost-saving option in every case, it consistently achieves superior effectiveness across all scenarios, indicating it may be a better choice for obtaining clinical efficacy, as corroborated by various scientific reports.^22–24^

### Strengths and limitations

We consider the major strength of our study lies in the fact that, to the best of our knowledge, it is the first cost-effectiveness study of telerehabilitation for ASs conducted through a clinical trial with a CG, thus representing an innovative study.

The most significant limitation was that a final or generic health measure was not accounted for (e.g., quality-adjusted life years, disability-adjusted life years, etc.), making it difficult to evaluate the opportunity cost (i.e., the benefits forgone) concerning other programs covered by the same budget.^25^

The second limitation was that indirect costs were not measured, which is known to substantially affect cost determination and cost-effectiveness analysis^8,15^; had these been included, it is possible that the intervention could have demonstrated even greater cost-saving benefits, as patients in the CG had more frequent consultations, resulting in higher indirect costs due to transportation or the need for an accompanying caregiver.

## Conclusion

Adding telerehabilitation to standard care for AS was cost-saving, achieving greater effectiveness at a lower cost. This is more evident when direct costs are considered together with the costs of work incapacity.

## Abbreviations Used

AS(s): Ankle sprain(s)
ADL: Activities of daily living
CER: Cost-effectiveness ratio
CG: Control group
DALYs: Disability-Adjusted life years
FAAM: Foot and ankle ability measure
ICER: Incremental cost-effectiveness ratio
IG: Intervention group
QALYs: Quality-Adjusted life years
SA: Sports activities
VAS: Visual analog scale
